# Effects of Dietary Quality on Vaginal Microbiome Composition Throughout Pregnancy in a Multi-Ethnic Cohort

**DOI:** 10.3390/nu16193405

**Published:** 2024-10-08

**Authors:** Corrie Miller, Kira Morikawa, Paula Benny, Jonathan Riel, Marie K. Fialkowski, Yujia Qin, Vedbar Khadka, Men-Jean Lee

**Affiliations:** 1 Department of Obstetrics and Gynecology, John A. Burns School of Medicine, University of Hawai’i, Honolulu, HI 96813, USA; kiraem@hawaii.edu (K.M.); jriel@hawaii.edu (J.R.); 2 National University of Singapore, Singapore 119077, Singapore; 3 Nutrition Support Shared Resource, University of Hawai’i Cancer Center, Honolulu, HI 96813, USA; mariekf@hawaii.edu; 4 Department of Quantitative Health Sciences, John A. Burns School of Medicine, University of Hawai’i, Honolulu, HI 96813, USA; yqin@hawaii.edu (Y.Q.); vedbar@hawaii.edu (V.K.)

**Keywords:** vaginal microbiome, healthy eating index, diet quality

## Abstract

**Background**: Vaginal *Lactobacillus* predominance is associated with improved vaginal health and reduced pregnancy complications. Little is known about how dietary quality may improve vaginal microbial composition or about dietary interventions that may promote *Lactobacillus* abundance. To understand the host factors affecting vaginal microbiota during pregnancy in a multi-ethnic cohort in Hawai`i. We hypothesize that better diet quality improves vaginal microbial composition, as represented by *Lactobacillus* abundance and depletion of anaerobic organisms. **Methods**: We compared comprehensive diet quality, as measured by the Healthy Eating Index-2015 (HEI-2015), to taxonomic classifications of bacteria present within the vagina. Participants of the four predominant ethnic groups in Hawai’i (Japanese, Filipino, Non-Hispanic White and Native Hawaiian) completed Quantitative Food Frequency Questionnaires (QFFQs) and collected vaginal swabs during each trimester. 16s rRNA amplicon sequencing (V2–V9 regions) was performed on vaginal samples. HEI-2015 scores and macro- and micronutrient intake were compared with the predominant species present using the Mann–Whitney-U test, PERMANOVA, and the Pearson correlation coefficient. A mixed-effects logistics regression model was used to predict the depletion of *Lactobacillus* species while accounting for confounding demographic factors. **Results**: Matched, longitudinal data for 40 participants demonstrated three predominant *Lactobacillus* species: *L. crispatus, L. iners,* and *L. gasseri*, with another subset of samples with anaerobic abundance. (Primarily, *Atopobium vaginae*, *Prevotella*, and *Gardnerella vaginalis*.) Non-Hispanic White participants had lower amounts of *Lactobacillus iners* compared to other racial and ethnic groups. HEI scores correlated with the chao index and observed species number primarily in the first trimester (r = 0.25, *p* < 0.05). Greater carbohydrate intake was associated with a higher abundance of *L. crispatus*, while lower carbohydrate intake trended towards more *L. iners* (0.056) and anaerobic species. **Conclusions**: Increased carbohydrate consumption and improved diet quality may be associated with beneficial vaginal microbial composition in pregnancy.

## 1. Introduction

The vaginal microbiome plays a crucial role in women’s health, as it consists of diverse bacteria that help maintain a balanced environment in the vagina. A healthy microbiome, dominated by beneficial bacteria like *Lactobacillus*, helps protect against sexually transmitted infections (STIs), bacterial vaginosis, and yeast overgrowth [1,2,3]. This occurs via *Lactobacillus*’ production of multiple defense compounds including lactic acid, which lowers the pH of the vagina and inhibits the growth of other invading pathogens [4,5]. The presence of *Lactobacillus* within the reproductive tract influences fertility and pregnancy outcomes [6,7]. Disruptions in this balance, known as dysbiosis, can lead to various reproductive health problems, making the study and understanding of the vaginal microbiome essential for improving women’s health and developing targeted treatments.

The establishment of microbial community patterns is complex and reflects a combination of social and environmental host factors, including race and ethnicity, parity, age, use of hormonal contraception, hygiene, and sexual practices [8,9,10]. Compared to the gastrointestinal microbiome, which is characterized as ‘healthy’ with a greater number of species present (termed “microbial diversity”, with higher diversity being healthier), a vaginal microbiome with beneficial properties has *less* diversity, typically with *Lactobacillus* species solely predominating most of the community. Previous studies have classified vaginal microbiome communities into five recognized “Community State Types” (CSTs) according to the most dominant species present within the community. These groups include: CST I (*L. crispatus*), CST II (*L. gasseri*), CST III (*L. iners*), and CST V (*L. jensenii*) [11,12,13]. CST IV is assigned to communities having no specific dominant species, often with many anaerobic species, known as the diverse group. 

There is limited research on the impact of dietary intake on the microbial composition of the reproductive tract, though it represents a modifiable factor that could potentially improve vaginal dysbiosis. While there is an abundance of knowledge regarding how dietary patterns and diet quality affect the gastrointestinal (GI) microbiome [14,15,16], few studies have investigated nutrition and the vaginal microbiome, particularly during pregnancy. Some individual foods that modulate the vaginal microbiome in pregnancy include dairy products, which are associated with increased *L. crispatus* in comparison to *L. iners* [17], and whole grains which are associated with an increase *Lactobacillus* dominance [18]. This association may occur through two different mechanisms. First, the gastrointestinal tract is within close anatomical proximity to the vagina, and just as ascending pathogens are known to enter the vagina and cause pathogenic infection within the reproductive tract (pelvic inflammatory disease or PID), beneficial microbes likely do the same. Secondly, specific micro- and macronutrients may affect hormonal or steroid production and glycemic regulation, thereby indirectly impacting the normal microbial community within the reproductive tract through prebiotic molecules [19]. 

One such example are carbohydrates, which may promote the storage of the important substrate, glycogen, for *Lactobacilli* metabolism [20]. High consumption of carbohydrates prior to pregnancy in an Italian cohort improved vaginal microbial composition and increased the amount of several beneficial metabolites (tryptophan, phenylpropionate, leucine, isoleucine, phenylalanine, O-acetylcholine, and sarcosine) in vaginal fluid [21]. Beyond specific micro- and macronutrients, a more holistic way to evaluate dietary consumption is through dietary patterns. The Healthy Eating Index (HEI) is a scoring metric that can determine overall diet quality and adherence to the Dietary Guidelines for Americans (DGA) [22]. Higher HEI scores have been associated with improved GIT microbial profiles [23] and several health outcomes [24], but few studies have looked at the relationship to the vaginal microbiome. 

Finally, ethnic-specific differences in the vaginal microbiome are documented across the world and seem to be an important host contributor to microbial composition [11,25,26,27]. Most of these studies have been in Non-Hispanic Black, Caucasian, and Hispanic women [26,28,29], and consistently show that Non-Hispanic White women are more likely to have vaginal microbial communities dominated by CST I, in comparison to Black and Hispanic women, who more commonly have CST IV and CST III, respectively [11]. Asian American, Native Hawaiian, and Pacific Islander (AANHPI) populations are underrepresented in this area, and typically aggregated together. Additional studies are warranted to further represent these racial and ethnic groups, as they may have unique environmental exposures, risk factors, and health outcomes related to the vaginal microbiome. 

As significant research efforts are underway to understand the impact of the vaginal microbiome on adverse pregnancy outcomes, such as preterm birth, pre-eclampsia, and fetal growth restriction [7,30,31,32,33], there are simultaneous investigations to understand modifiable and translatable interventions, such as diet, to modulate the vaginal microbiome. This study aims to understand the impact of comprehensive diet quality (as measured by HEI) on vaginal microbial composition and diversity across gestation in a cohort of low-risk pregnant individuals, in an understudied and underrepresented multi-ethnic cohort. We hypothesized that vaginal CSTs would differ among pregnant persons of various ethnicities in Hawai’i, and that better diet quality would lead to a higher abundance of beneficial *Lactobacilli* species, and depletion of anaerobic bacteria within the vagina.

## 2. Materials and Methods

### 2.1. Study Subjects and Recruitment

**Figure 1:**
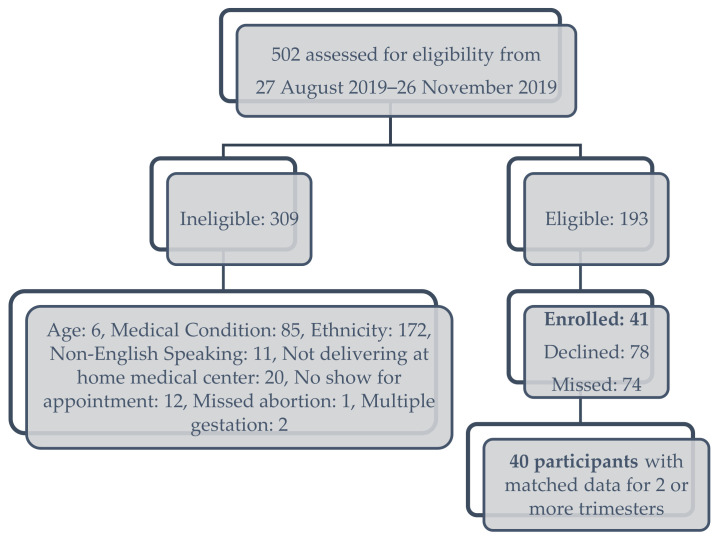
CONSORT diagram of participant assessment for eligibility and enrollment.

This longitudinal cohort study was approved by the Western Institutional Review Board in compliance with the Hawai‘i Pacific Health protocol. The protocol has previously been described [34]. Participants were recruited from August to November, 2019 from the 4 most common racial and ethnic groups in Hawai‘i—Japanese, Filipino, Native Hawaiian, and non-Hispanic White, prior to 14 weeks gestation. This sampling method was used to ensure equal representation across race and ethnicity. Inclusion criteria were: women aged 18–45 years old, primarily English-speaking and English-literate, self-identified as having one or both parents that identified as their reported heritage of 3 of the 4 main ethnicities. Native Hawaiians of any “percentage” of ethnicity were eligible, while participants that identified as mixed-race and -ethnicity were excluded. Other exclusion criteria were: plans to move out of the area prior to delivery, a plan to deliver at another hospital other than the medical center of the research team, multiple gestation, pre-existing diabetes or hypertension, heart disease, chronic renal disease, systemic lupus erythematosus, hypothyroidism, history of bariatric surgery, history of an eating disorder, or inflammatory bowel disease, and women who were currently incarcerated. Figure 1 demonstrates the representative CONSORT diagram of participant enrollment. 

### 2.2. Data Collections

Participation included completing the Multiethnic Cohort Quantitative Food Frequency Questionnaire (MEC QFFQ) [35] three times, once during each trimester, and also collecting microbiome samples via self-collected vaginal swab at the same time points in each trimester. 

#### 2.2.1. Quantitative Food Frequency Questionnaire

The MEC QFFQ was developed and validated in a large population from 1993 to 1996 in Hawai’i and California [36]. The tool has proven effective in associating diet quality with several health outcomes [37,38]. While being developed, participants could identify as Hispanic, Japanese American, Native Hawaiian, Non-Hispanic Black, and Non-Hispanic White or Other. For respondents that identified as “other”, the majority (2/3) identified as Filipino. The average daily intake of energy and nutrients was calculated from 24 h recalls into a food composition table. The 182-item QFFQ includes 85 specific food items uniquely associated with the traditional diets of a particular group irrespective of their contribution to intake, such as poi, taro, spam, tofu, salted fish, miso soup, saimin, and fermented foods.

The data extracted from the Food Frequency Questionnaire were analyzed by the University of Hawai`i Cancer Center Nutrition Shared Support Resource. The resource provides information on 54 nutrients from food, energy, macronutrients, and 24 nutrients from supplements, which has been carefully calculated after several validation calibration studies [35,39]. Standard analysis also provides four Diet Quality Scores (DQS): The Alternative Healthy Eating Index (AHEI), the Alternative Mediterranean Diet (aMED), the DASH Diet score, and the Healthy Eating Index-2015 (HEI-2015) [40] (Table 1). The indexes have been studied in pregnant populations to correlate with various pregnancy outcomes. The HEI is the most studied, and has been used to link diet patterns with gestational weight gain, preterm labor, pre-eclampsia, and neonatal birth weight [24,41,42,43,44], and thus was the primary index compared to microbiome data. Higher HEI scores represent better diet quality and better adherence to the Dietary Guidelines for Americans (DGA). HEI-2020 was not available at the time of performing this analysis. 

#### 2.2.2. Vaginal Swab Collection

The first QFFQ and bacterial swab collection were completed at the time of enrollment, at 11–13 weeks’ gestation. The second collection occurred in the second trimester at the time of the scheduled office fetal ultrasound at 18–20 weeks’ gestation. Third trimester samples were collected at 34–36 weeks’ gestation at home. Participants were provided with a Copan e-swab for self-collection, and instructed to insert the swab into the mid-vagina and rotate for 15 s. Specimens were either collected in the office and given to research staff or sent via postal mail within 24 h of collection. Specimens were immediately frozen at −80 °C until further processing. 

#### 2.2.3. Demographic Data and Pregnancy Outcome Data Extraction

Demographic data and pregnancy information were collected from electronic medical records, including parity, obesity (body mass index >30 kg/m^2^) at first prenatal visit, excess gestational weight gain at the end of pregnancy (as defined by Institute of Medicine Guidelines according to body mass index) [45], gestational age at delivery, birth weight, and mode of delivery. Pregnancy complications, including gestational diabetes, pregnancy-associated hypertension, preterm labor, and fetal growth restriction, were extracted from the mother’s medical record and neonatal complications, including Neonatal Intensive Care Unit (NICU) admission, hyperbilirubinemia, and need for respiratory support, were also acquired from the neonatal medical record. 

### 2.3. Microbiome Sequencing

DNA isolation was performed using the AllPrep DNA/RNA Extraction Kit (Qiagen, Hilden, Germany). ThermoFisher Scientific 16S rRNA primers were then used to create bacterial DNA libraries for sequencing according to the manufacturer’s instructions. Metagenomic sequencing was carried out on the Ion Genestudio S5 Sequencer (ThermoFisher Scientific). V2-9 primers were used to amplify the hypervariable regions of the 16S rRNA gene from bacteria. Reads were matched to the Greengenes v13.5 and MicroSEQ ID v3.0 reference databases. Using the Ion Torrent analysis platform, sequences were aligned, and operational taxonomic units (OTUs) were classified at family, genus, and species levels. To account for differences in read counts between samples, raw abundance values were normalized by subsampling to 10,000 reads per sample, focusing on the species-level OTU table. Samples with fewer than 10,000 total reads were excluded from the final dataset. The microbial alpha diversity of maternal samples for each trimester was calculated using the Chao1, Shannon, and Simpson indexes. To ensure robustness, α-diversity indexes following rarefaction were computed using the average of 10 rarefied values at a sequence depth of 15,927 reads. Beta diversity profiles were evaluated through principal component analysis (PCA) based on the Bray–Curtis distance matrix. 

### 2.4. Data Analysis

The characteristics of the participants were summarized by median and standard deviation for continuous variables (or interquartile range for non-normally distributed data), frequencies, and percentages for categorical variables. The two-tailed Student’s *t* test, ANOVA, or χ2 test were used to test the differences in these variables, respectively. Non-parametric tests (Mann–Whitney U and Kruskal–Wallis test) were applied for non-normally distributed data. Repeated measures ANOVA with a multiple comparison test via Bonferroni as post hoc analysis was used to compare alpha diversity (Shannon, Chao 1, Simpson) indexes with demographic characteristics where appropriate, such as ethnicity and obesity aggregately among the trimesters. CST was assigned by determining the organism (species) with the highest absolute abundance within each sample. Beta diversity profiles were analyzed with principal component analysis (PCA) among each ethnic group, trimester, and according to dietary pattern adherence (HEI dietary pattern quartiles) after a Bray–Curtis distance matrix was developed, and a PERMANOVA test used to evaluate dispersion between centroids of maternal characteristics. The percentage of carbohydrate to protein to total fat consumption was compared to investigate the effect of macronutrient consumption on *Lactobacillus* species predominance, as well as all of the top 10 most abundant species. The specific effect of carbohydrate intake was investigated on CST and overall species abundance. Participants were categorized into “high” vs. “low” carbohydrate intake, defined as those above and below the median intake of carbohydrates daily, defined as a percentage of caloric intake (<49% = low, 49% = high). Mean micro- and macronutrient consumption were compared among those with complete depletion of *Lactobacillus* species vs. those who had some predominance of *Lactobacillus* with non-parametric tests. All data analyses were performed using R Studio version 2024.04.2 (http://www.r-project.org/ (accessed on 2 February 2024) and a two-tailed *p*-value of less than 0.05 was regarded as statistically significant.

## 3. Results

### 3.1. Study Group Demographics

Forty-one participants were recruited, and there were matched, longitudinal data (microbiome analysis with QFFQ measurement in each trimester) available for 40 participants (Figure 1). The baseline demographics and QFFQ information of the cohort by race and ethnicity are presented in Table 2. Aggregate dietary pattern scores differed among the four groups, with Native Hawaiians having the highest diet quality. Native Hawaiian participants consumed a higher proportion of carbohydrates, compared to the other racial and ethnic groups; fat and protein consumption did not differ among the four groups. 

### 3.2. 16S Metagenomic Data

**Figure 2:**
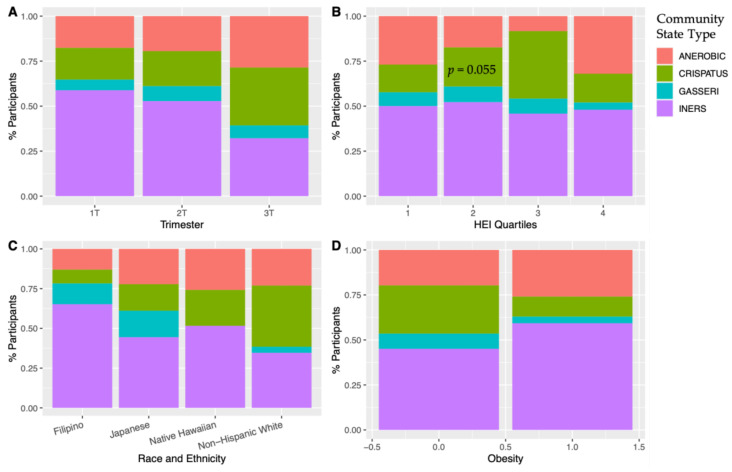
Community state type (CST) according to (**A**) trimester, (**B**) HEI quartile [1st quartile = lowest diet quality, 4th quartile = highest diet quality], (**C**) race and ethnicity, and (**D**) obesity. There was an increase in *Lactobacillus cristpatus* in the 3rd trimester. Non-Hispanic White participants had a predominance of *Lactobacillus crispatus*. In a mixed-effects multivariate model with the displayed covariates, comparing anerobic species predominance vs. all others, HEI approached significance (*p* = 0.055) with the 3rd quartile of HEI scores having the lowest predominance of anerobic species.

Read counts averaged at 65,749 reads per sample. Predominant CST by race and ethnicity, across trimester, obesity status, and HEI quartiles is presented in Figure 2. *Lactobacillus crispatus* was most prevalent in Non-Hispanic White participants, while *L. iners* was predominant in the other three groups. No other significant differences are seen among diet quality or obesity. While the 3rd quartile of the HEI score had low amounts of anaerobic OTUs with a higher predominance of *L. crispatus*, the 4th quartile a had much higher anaerobic predominance, negating a linear relationship with CST and *Lactobacillus* predominance and higher diet quality in this analysis. 

**Figure 3:**
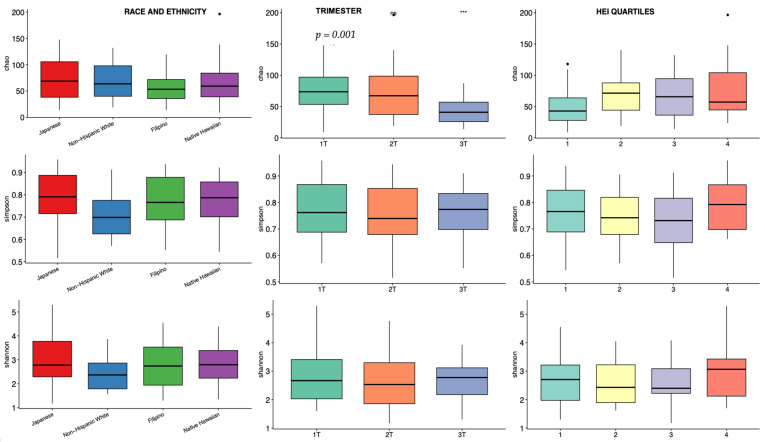
Vaginal alpha diversity across three indices: Chao index (top row), Simpson index (middle row), and Shannon index (bottom row), stratified by race and ethnicity (Column 1), trimester (Column 2), and Healthy Eating Index (HEI) quartiles (Column 3). The first quartile represents the lowest diet quality, and the fourth quartile represents the highest. A significant decrease in the Chao index is observed in the third trimester compared to the first and second trimesters (*p* = 0.001, indicated by ***). “ns” indicates no significant differences. Colored box plots represent the respective categories for each variable, while the dots indicate potential outliers in the data.

**Figure 4:**
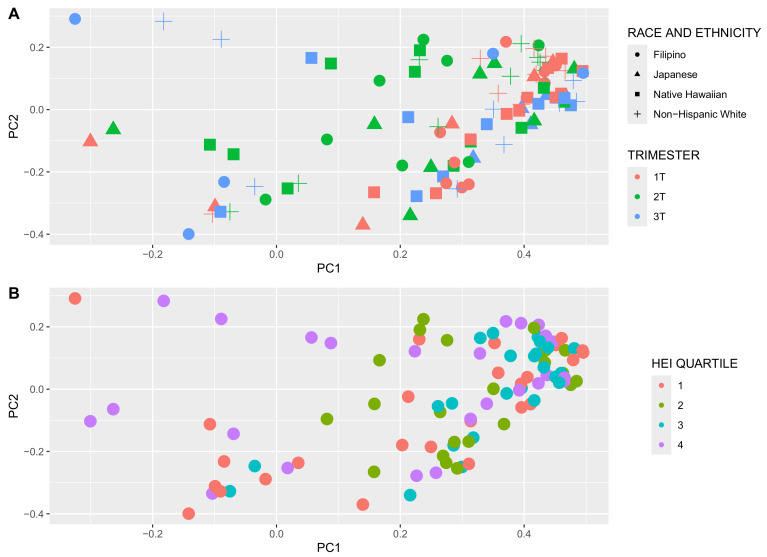
Beta diversity principal component analysis (Bray–Curtis distance), (**A**) according to race and ethnicity, trimester, and (**B**) Healthy Eating Index (HEI) quartile (1 = lowest quality, 4 = highest quality). Trimester demonstrates some disparate groupings in the first vs. second trimesters (green vs. red in panel **A**). The other examined maternal covariates did not group closely together.

**Figure 5:**
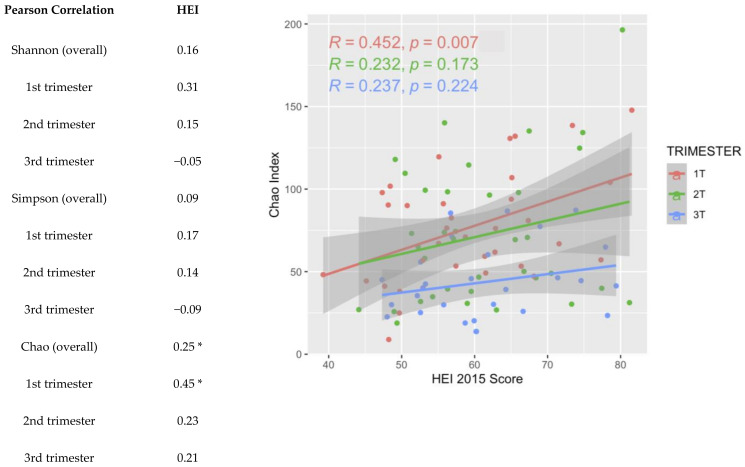
R coefficients of Healthy Eating Index score (HEI-2015) and alpha diversity metric (Shannon, Simpson, or Chao) via Pearson correlation. * Chao was significantly correlated with dietary quality primarily in the first trimester (*p* values < 0.05).

Alpha diversity, a metric of individual sample diversity, is shown according to race and ethnicity, trimester, and HEI quartiles in Figure 3. Reduced diversity within a vaginal microbial community is generally considered more beneficial, associated with to *Lactobacillus* predominance. Among the three alpha diversity metrics compared, there were no differences in alpha diversity according to race and ethnicity or adherence to HEI. There was a significant decrease in Chao index in the 3rd trimester compared to 1st and 2nd trimesters; Shannon and Simpson indexes were not different among the trimesters. Figure 4 demonstrates beta diversity (how different participant samples are from one another) by PCA plot for the same covariates using Bray–Curtis distance matrices. Each sample is represented by a point on the plot; those samples with more phylogenetic similarity are grouped more closely together, while samples that are dissimilar are farther apart. No significant grouping was identified by race and ethnicity or HEI quartile, via PERMANOVA analysis of centroid dispersion. Linear correlation via Pearson correlation was investigated according to average HEI score throughout all three trimesters, and according to trimester. The only significant finding was with the Chao index in the first trimester (Figure 5). 

**Figure 6:**
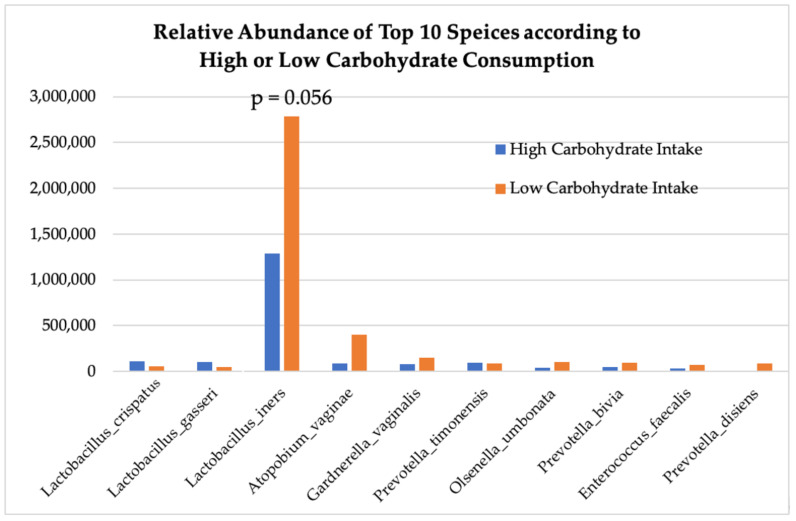
Absolute abundance of operational taxonomic units (OTUs) at species level according to high or low consumption of carbohydrates.

**Figure 7:**
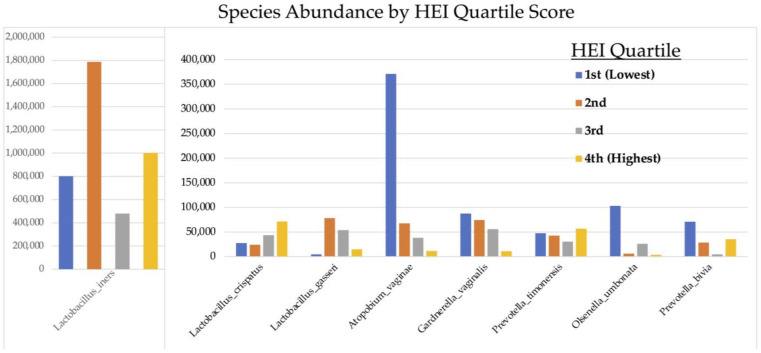
Absolute abundance of operational taxonomic units (OTUs) at species level according to Healthy Eating Index (HEI) quartiles.

Figure 6 demonstrates that carbohydrate consumption trended towards having a significant effect on *Lactobacilli* abundance throughout the entire cohort, with higher carbohydrate intake associated with greater abundances of *L. crispatus* and *L. gasseri* and lower carbohydrate consumption associated with greater abundances of *L. iners* (*p* = 0.056) and aerobic species: *Gardnerella vaginalis, Prevotella timonensis,* and *Atopium vaginae* (Figure 7). HEI adherence also showed trends with species type: higher HEI scores were associated with higher levels of *L. cristpatus*, and lower HEI score was associated with more anaerobic-type bacteria including *Gardnerella vaginalis* and *Atopium vaginae* (Figure 6), although differences in absolute read counts were not significant. 

Analysis of 54 macro- and micronutrients showed no differences with repeated measures ANOVA among the four CST groups, or bivariate comparison of complete *Lactobacilli* depletion. Finally, a mixed-effects logistic regression model was used to evaluate maternal predictors of *Lactobacilli* depletion and anaerobic abundance. Among race and ethnicity, trimester, age, HEI Score, carbohydrate intake, and obesity status, no significant predictors were identified.

## 4. Discussion

Among the many factors that influence vaginal microbiota including hormonal fluctuations, body habitus, geography, and ethnicity [46,47,48,49,50], dietary consumption is a modifiable intervention to target health. This is the first study to associate comprehensive diet quality as measured by HEI with the vaginal microbiome in pregnancy. We found higher HEI was associated with greater abundance of *L. crispatus* and *gasseri*, organisms known to have beneficial properties. Anaerobic organisms (*Atopobium vaginae)* and *L. iners* species were more predominant in pregnant persons with lower HEI scores. These species have been associated with less stable microbial communities in the vagina, and subsequently, poor pregnancy outcomes, vaginal infections, and higher acquisition of sexually transmitted infections [51,52,53,54]. We also observed an increase in carbohydrate intake correlated with *Lactobacillus* abundance, primarily *L. crispatus* and *gasseri* growth, while lower carbohydrate consumption was associated with an increase in *L. iners* and anaerobic growth. 

The effects of HEI and carbohydrate intake on vaginal microbial composition are understudied. Most nutritional research on the vaginal microbiome has investigated the effects of dairy products and probiotics [48,55,56], due to the presence of *Lactobacilli* cultures within yogurts and other fermented food products [57,58]. In a North Carolina cohort, it was found that higher consumption of dairy was associated with *Lactobacillus crispatus* predominance, as was fruit, vitamin D, fiber, and yogurt consumption in the second trimester of primarily Black women [17]. 

While the introduction of beneficial bacteria may help change microbial community composition in the short term, it is important to introduce prebiotics to support long-term structural change. Glycogen is one such important substrate for *Lactobacilli*, and increasing the storage of glycogen through starch and carbohydrate intake may promote its abundance [59]. Sun et al. performed a dietary intervention in 103 pregnant women and randomized them to increase their consumption of whole grains vs. refined grains by 75%. They noted a greater reduction in alpha diversity, with increasing *Lactobacillus* dominance, after the intervention, in those consuming higher amounts of whole grains [18]. Complete CST characterization was not performed in this study. 

When making dietary recommendations, it is important to have a comprehensive approach, instead of focusing on one particular micro- or macronutrient. Adherence to dietary patterns has been shown to be beneficial in improving gut microbial health [15,23,60]. Further understanding of the impacts of vaginal microbial health is needed. Noormohammadi et al. noted that, outside of pregnancy, the highest tercile of HEI scores decreased the odds of acquiring bacterial vaginosis, as measured by Amsel criteria [61]. Our study is the first to compare this diet quality index to specific vaginal microbial taxonomic classifications in pregnant individuals.

Other maternal host factors interact with diet, including race and ethnicity. Diet is a significant component of cultural expression and can vary greatly between race and ethnic groups. Several characterizations of the vaginal microbiome during pregnancy exist in Non-Hispanic Black [62], Non-Hispanic White [25], Hispanic [63], and Asian populations [27]. None of these studies have considered the effects of nutrition on vaginal microbial composition. The Pacific is home to a uniquely diverse ethnic and cultural population with Pacific Islanders representing over 20 ethnic subgroups alone [64]. Asians and Native Hawaiian and Pacific Islander people have been noted to comprise the fastest expanding groups in the United States [65], but are typically aggregated together in demographic data [66]. There is limited data about the vaginal microbiome during pregnancy and reproduction. This study provides the first description of vaginal microbial composition and its interplay with diet quality in a prospective cohort in the Pacific. 

Finally, the natural hormonal shifts of estrogen and progesterone during pregnancy are also known to contribute to microbial composition [67,68]. Previous studies have demonstrated a trend toward less microbial diversity and a predominance of *Lactobacilli* species in the third trimester [69]. This is likely due to the hormonal and physiologic changes in pregnancy [70], as estrogen reaches peak levels in the third trimester and is known to increase the amount of free glycogen in vaginal epithelial cells [71]. The rise in estrogen during pregnancy may offer an explanation for the mechanism by which alpha diversity decreases throughout pregnancy, as was also verified in our cohort, indicating an increase in *Lactobacilli* growth which is associated with improved outcomes [71].

The limitations of this study design include a smaller sample size, in a primarily healthy cohort of pregnant individuals, which makes the findings possibly less generalizable to those with pregnancy complications. We did not measure serum or vaginal excretion of hormone levels throughout pregnancy in association to the vaginal microbiome, as well as micronutrient or macronutrient interactions with vaginal microbial species, and such investigations should be considered for future research. Additional studies are required to confirm our findings and see how a dietary intervention during pregnancy would be able to affect vaginal microbiome dysbiosis and the associated poor pregnancy outcomes such as preterm labor. Overall, this study elucidates the positive relationship between carbohydrate consumption and a higher HEI with *Lactobacilli* growth, demonstrating strategies to encourage the abundance of beneficial *Lactobacilli* species growth within the vagina. Further research is needed to confirm the hypothesis of dietary quality impact on the vaginal microbiome, and how this could improve reproductive health outcomes.

## 5. Conclusions

This is the first study to show the longitudinal changes in the vaginal microbiome during pregnancy associated with diet quality in a multi-ethnic cohort of pregnant people in the Pacific. Adherence to the dietary guidelines for Americans, as measured by HEI, appears to promote acquisition of beneficial vaginal microbial composition.

## Figures and Tables

**Table 1:** Healthy Eating Index-2015 components, point values, and standards for scoring. Adapted from Krebs-Smith et al., 2018 [40]. ^a^ PUFAS = polyunsaturated fatty acids, ^b^ MUFAS = monounsaturated fatty acids, ^c^ SFAs = saturated fatty acids.

Component	Maximum Points	Standard for Maximum Score	Standard for Minimum Score
*Points given for adequacy of consumption*			
Total Fruits	5	≥0.8 c equivalents/1000 kcal	No fruit
Whole Fruits	5	≥0.4 c equivalents/1000 kcal	No whole fruit
Total Vegetables	5	≥1.1 c equivalents/1000 kcal	No vegetables
Greens and Beans	5	≥0.2 c equivalents/1000 kcal	No dark green vegetables or beans and peas
Whole Grains	10	≥1.5 oz equivalents/1000 kcal	No whole grains
Dairy	10	≥1.3 c equivalents/1000 kcal	No dairy
Total Protein Foods	5	≥2.5 oz equivalents/1000 kcal	No protein foods
Seafood and Plant Proteins	5	≥0.8 oz equivalents/1000 kcal	No seafood or plant proteins
Fatty Acids	10	(PUFAs ^a^ + MUFAs ^b^)/SFAs ^c^ ≥ 2.5	(PUFAs + MUFAs)/SFAs ≤ 1.2
*Points given for moderation of consumption*			
Refined Grains	10	≤1.8 oz equivalents/1000 kcal	≥4.3 oz equivalents/1000 kcal
Sodium	10	≤1.1 g/1000 kcal	≥2.0 g/1000 kcal
Added Sugars	10	≤6.5% of energy	≥26% of energy
Saturated Fats	10	≤8% of energy	≥16% of energy

**Table 2:** Baseline demographics according to self-identified race and ethnicity.

	Filipino *n* = 10	Japanese *n* = 10	Native Hawaiian *n* =10	Non-Hispanic White *n* = 10	*p*-Value
Age (median, [SD])	25 [5.03]	34 [5.02]	31 [6.03]	32 [5.55]	NS
Parity (median)	0	0.5	0	1	
Obesity ^1^	1	3	6	3	
Excess Gestational Weight Gain ^2^	0	0	6	3	
Mode of Delivery					
Cesarean Delivery	3	3	4	0	
Vaginal Delivery	7	7	6	10	
Pregnancy Complications					
Gestational Diabetes	0	1	2	1	
Preeclampsia	1	5	2	1	
Spontaneous Preterm Labor	1	0	0	0	
Healthy Eating Index Score (median) [Interquartile Range]	55.71 [52.59, 57.41]	60.38 [53.08, 67.41]	65.38 [53.70, 75.85]	60.68 [55.38, 65.38]	0.032 ^a^
Macronutrient Intake					
Percent of Energy from Protein	16.24 (2.11)	16.29 (2.32)	16.37 (2.90)	15.85 (2.01)	NS
Percent Energy from Carbohydrate	50.15 (6.35)	45.60 (6.29)	52.33 (6.94) ^b^	47.19 (5.19)	0.017
Percent Energy from Fat	34.78 (4.88)	37.67 (5.63)	33.11 (5.32)	36.47 (3.88)	NS

^1^ Obesity = body mass index >30 kg/m^2^ at the first prenatal visit. ^2^ Excess gestational weight gain as defined by the Institute of Medicine recommended number of lbs relative to body mass index; NS = not significant; ^a^ Native Hawaiian > Filipino; ^b^ Native Hawaiians had a greater consumption of carbohydrates compared to Japanese (repeated measures ANOVA).

## Data Availability

The data described in the manuscript, code book, and analytic code will be made publicly and freely available without restriction following the acceptance of our manuscript for publication. These datasets will be deposited into appropriate databases including the NCBI Gene Expression Omnibus (GEO) database, the NCBI Short Read Archives (SRA), MicrobiomeDB, and other relevant databases and made freely available to investigators at academic institutions worldwide. The authors would like to acknowledge Carol Boushey, PhD for her contributions to the original study design and analysis through the Nutrition Support Shared Resource at the University of Hawai’i Cancer Center.
